# Synergistic Effects of Cryptotanshinone and Senkyunolide I in Guanxinning Tablet Against Endogenous Thrombus Formation in Zebrafish

**DOI:** 10.3389/fphar.2020.622787

**Published:** 2021-01-14

**Authors:** Jun Li, Hao Liu, Zhenzhong Yang, Qingqing Yu, Lu Zhao, Yi Wang

**Affiliations:** ^1^ Pharmaceutical Informatics Institute, College of Pharmaceutical Sciences, Zhejiang University, Hangzhou, China; ^2^ The Department of Medicine, Chiatai Qingchunbao Pharmaceutical Co., Ltd., Hangzhou, China; ^3^ State Key Laboratory of Component-Based Chinese Medicine, Tianjin, China

**Keywords:** thrombosis, coronary artery disease, senkyunolide I, cryptotanshinone, oxidative stress, zebrafish, multimodal identification

## Abstract

Thrombosis is a key pathological event in cardiovascular diseases, and is also the most important targeting process for their clinical management. New drug development in thrombosis treatment is still in great demand. According to the traditional Chinese medicine (TCM) theory, thrombosis belongs to the syndrome of blood stasis. *Salvia miltiorrhiza* Bunge and *Ligusticum striatum* DC*.* are two common TCM herbs with long-term documented function in promoting blood circulation and inhibiting thrombosis, especially when used together. Guanxinning Tablet, a modern Chinese drug which contains extracts of the two herbs, also showed strong therapeutic effects in coronary heart disease. However, the pharmacological mechanism is still lacking for the compatibility of the two herbs. Here, through zebrafish-based *in vivo* fluorescence screening, we demonstrated the synergistic effects between *S. miltiorrhiza* Bunge and *L. striatum* DC. in regulating endogenous thrombosis. Moreover, combined with high-resolution mass spectrometry, the main compounds of the botanical drugs were analyzed and screened in our model system. Interestingly, cryptotanshinone and senkyunolide I, two representative compounds, respectively derived from the two herbs, also showed synergistic antithrombotic effects. Further analysis suggested that they may regulate thrombi formation at different levels *via* multiple signaling pathways, including oxidative stress, platelet activation and coagulation cascade. Taken together, our findings provided solid biological supports toward the drug compatibility theory of TCM, and suggested cryptotanshinone and senkyunolide I as promising drug candidates in thrombosis management.

## Introduction

Thrombosis is the key pathological process in coronary artery disease (CAD), which remains the leading cause of global DALYs for 30 years. The breakage of atherosclerosis plaque may lead to artery blockage, which triggers heart attack, stroke or sudden death. Venous thromboembolism (VET) is also a common type of cardiovascular disease, which usually develops in patients with major trauma. Besides, multiple epidemiological studies reported increased incidence of VET in patients with a history of arterial cardiovascular events or with family history of myocardial infraction (Braekkan et al., 2008; Sørensen et al., 2009). Although whether arterial and venous thrombosis are linked processes is still controversial, the comorbidity of VET in coronary artery diseases makes the treatment even more challenging.

As plaque rupture-initiated platelet activation is usually regarded as the first step in an acute coronary event, antiplatelet therapies are mainly used in the clinical management of CAD. Aspirin, for example, is a well-established antiplatelet drug in the secondary prevention of CAD patients, which irreversibly inhibits cyclooxygenase-1 (COX1), also named as prostaglandin-endoperoxide synthase 1 (PTGS1), and suppresses the biosynthesis thromboxane A2 (TxA2) (Depta and Bhatt, 2015). Nevertheless, the intravascular imaging-based PROSPECT study only detected <5% of thin-capped fibroatheroma actually ruptured and provoked an acute coronary event during a 3.4 years observation period (Stone et al., 2011), suggesting that plaque rupture may not be the only cause. On the other hand, the role of superficial erosion of plaques was gradually recognized in CAD, which may involve multiple processes including endothelial damage and innate immune activation (Libby et al., 2019). Therefore, conjunctive therapeutic approaches, including antiplatelet agents, anti-inflammation therapies, immunoregulators, and anticoagulant drugs, may bring more benefits for CAD patients.

Traditional Chinese medicine (TCM) has millennial practical experiences in using a combination of medicinal herbs in the treatment of various diseases. For example, according to the TCM compatibility theory, the combined usage of *Salvia miltiorrhiza* Bunge (*S. miltiorrhiza*) and *Ligusticum striatum* DC. (*L. striatum*) leads to enhanced efficacy in promoting blood circulation and resolving hemostasis, and was traditionally applied in the treatment of patients with typical CAD symptoms. Indeed, Guanxinning Tablet (GXNT), which is composed of extracts from the above two plants, showed significant effects in the clinical management of CAD. A multicenter RCT study in 160 CAD patients demonstrated greatly enhanced performance in the exercise tolerance test, along with reduced angina pectoris symptoms and improved ECG results in the GXNT group, compared with the placebo group (Sun et al., 2019). Nevertheless, due to the complicate composition in naturally derived materials, whether or how the two consisting herbs of GXNT cooperatively regulate CAD is still largely unclear.

The purpose of the current study is to examine the role of GXNT, and the cooperative effects of its main components, in the treatment of thrombosis. In order to dynamically observe the endogenous blood circulation, we took advantage of the zebrafish model system. The movement of fluorescent-labeled erythrocytes can be conveniently detected in *Tg* (*LCR:eGFP*) transgenic zebrafish (Ganis et al., 2012). Besides, the capability of percutaneous oxygen absorption in early-stage zebrafish embryos allows them to survive for a long time even without functional blood circulation, which provides valuable time window for the efficacy evaluation of anti-thrombotic agents. The zebrafish thrombosis model was induced by phenylhydrazine (PHZ), which is known to cause oxidative damage and endothelial dysfunction (Jain, 1985; Sato et al., 2015) and used as a thrombosis inducer in different animal models (Sato et al., 2009; Zhu et al., 2016). Combining the methods of high-resolution mass spectrometry and the high-efficient *in vivo* screening system, we identified major chemical compounds in GXNT, and evaluated their anti-thrombotic effects in the zebrafish model. A positive combination effect between cryptotanshinone and senkyunolide I, two representative compounds from the two GXNT herbs, was identified in the regulation of thrombogenesis, which possibly functions through mediating oxidative stress, platelet activation and coagulation cascade. These findings provided biological evidences toward the pharmacological mechanism of GXNT in CADs, and suggested novel drug combinations for antithrombotic therapy.

## Materials and Methods

### Animal Care Ethics

All zebrafish experiments were conducted according to the guidelines of Animal Ethics Committee of the Laboratory Animal Center, Zhejiang University.

### Zebrafish Husbandry

Wildtype TU strain, *Tg* (*LCR: EGFP*) (Ganis et al., 2012) and *Tg* (*CD41: EGFP*) (Lin et al., 2005) transgenic zebrafish were all obtained from the Laboratory Animal Center of Zhejiang University. Zebrafish were maintained following standard protocols (Westerfield, 2007). E3 medium (0.29 g/l NaCl, 0.013 g/l KCl, 0.048 g/l CaCl_2_ 2H_2_O, 0.082 g/l MgCl_2_ 6H_2_O, pH 7.2) was used as the embryo medium. Embryos were obtained through natural spawning.

### Chemicals and Reagents

PHZ (G1324044) was purchased from Aladdin company of Shanghai, China. 1-phenyl 2-thiourea (PTU, P7629) and Ethyl 3-aminobenzoate methanesulfonate (Tricaine, E10521) were purchased from Sigma-Aldrich company of United States. DCFH-DA (MA0219) and Aspirin (MB1790) were purchased from Dalian Meilun Biotechnology Company, China. MDA (S0131) was purchased from Beyotime Company of Shanghai, China. Cryptotanshinone was purchased from Winherb Medical Technology Company of Shanghai, China. Salvianolic acid B (B20261), Danshensu (B20254), Rosmarinic acid (B20862), Ferulic acid (B20007), Senkyunolide I (B21463) were purchased from Shanghai Yuanye Biotechnology Co., Ltd. (Shanghai, China). The purity of the six compounds are all greater than 98%.

### Preparation of GXNT and Herbal Extracts

GXNT and herb extracts were manufactured by Chiatai Qingchunbao Pharmaceutical Co. Ltd. (Hangzhou, China, Batch No. 201912), according to the Standard of National Medical Products Administration of China (YBZ00342016). Briefly, the rhizome of *S. miltiorrhiza* Bunge, *L. striatum* DC. or the two herbal compositions at 1:1 wt ratio (for GXNT), was collected and then extracted with water twice. The extracts were combined and concentrated, and then precipitated by adding alcohol. The insoluble substance was removed through a filter, and the filtrate was further concentrated to a relative density between 1.30 and 1.35 at 50°C. Finally, the concentrated extracts were dried to powder in a vacuum drying oven.

### UPLC-HRMS Analysis

GXNT was analyzed on a UPLC (Waters, Milford, MA, United States) coupled with a Q-Exactive™ Focus mass spectrometer (Thermo Scientific, Bremen, Germany). Chromatographic separation was performed on a Waters BEH C18 (100 mm × 2.1 mm, 1.7 μm) with acidified water with 0.15% formic acid and acetonitrile with 0.15% formic acid as mobile phases A and B at 30°C. The following solvent gradient was adopted: 0 min, 2% B; 8 min, 98% B. The flow rate was set at 0.6 ml/min, and a volume of 6 μl was injected for the analysis. The parameters for mass spectrometer were as follows: curtain gas, 30 psi; Gas 1 (N2), 55 psi; Gas 2 (N2), 55 psi; ion spray voltage, 3.5 kV (positive mode); 2.5 kV (negative mode) temperature, 350°C; scan range, m/z 120–1,500.

### Molecular Networking

Mass data were processed by MZmine2 and corresponding molecular networking was created according to the online workflow at GNPS (http://gnps.ucsd.edu) (Wang et al., 2016) with a parent mass tolerance of 0.02 Da and an MS/MS fragment ion tolerance of 0.02 Da, the minimum cluster size of one, run MScluster and filter precursor window tools were turned off. The network created with cosine score above 0.7 and more than four matched peaks. The spectra in the network were then searched against GNPS spectral libraries. The molecular networking data were visualized using Cytoscape (ver. 3.7.2).

### Zebrafish Thrombosis Modeling and Drug Treatment

Adult, sexually mature zebrafish were used to generate embryos. Healthy fertilized embryos were selected by microscopic examination and then raised in embryo medium containing PTU (0.2 mM) at 28°C for 2 days to inhibit melanization as described before (Karlsson et al., 2001). Zebrafish embryos of 60 h post fertilization (60 hpf) were collected into a 12-well microplate (12–15 fish per well), and were treated with aspirin (30 μg/ml), GXNT (1 mg/ml), *S. miltiorrhiza* (312 μg/ml), *L. striatum* (463 μg/ml), compounds (Cryptotanshinone 1 μg/ml, Ferulic Acid 25 μg/ml, and 50 μg/ml for all the other compounds), or compound combination (Cryptotanshione 1 μg/ml + Senkyunolide I 50 μg/ml) for 24 h. The concentration of GXNT was determined by its toxicity in zebrafish embryos, and the concentration of herbs was calculated based on their respective contents in the GXNT extract. For all chemical compounds, their concentrations were determined combinedly according to their toxicity in zebrafish embryos and solubility in the embryo medium. A sub-maximal tolerable dosage without inducing any general developmental defects was chosen for each compound. Dimethyl sulfoxide (DMSO, 0.1% v/v final concentration) was used as negative control. At the 84 hpf stage, the larvae were washed three times with fish water, and treated with PHZ (0.75 µM) for another 12 h. After PHZ treatment, the embryos were anesthetized and transferred into 96-well microplate (one fish per well) and images were acquired under Leica DMI 3000B inversed microscope system (Leica Microsystems Inc., United States).

### Quantification of Blood Flow and Circulating Platelets

The blood flow and circulating platelets were analyzed in *Tg* (*LCR:eGFP*) and *Tg* (*CD41:eGFP*) transgenic embryos, respectively. Embryos were anesthetized in 0.016% Tricaine during imaging. A video was taken at a speed of 50 frame/s for each embryo. For image analysis, a representative measurement area of the vein is manually located and the number of red blood cells passing through the vessel at a given point in all 50 frames is manually counted for each embryo. At least eight embryos were examined for treatment condition. Platelets were counted in the same way as erythrocytes, and the number of platelets passing through the blood vessel of representative sites was calculated in all 50 frames.

### Flow Cytometry

3.5 dpf *Tg* (*LCR:eGFP*) embryos with respective treatment were collected with at least 30 embryos in each group. FACS sorting of red blood cell (GFP positive) was performed on ACEA NovoCyteTM (ACEA Biosciences, United States). All steps were performed at 4°C.

### ROS Measurement

Zebrafish embryos were incubated with 10 μM DCFH-DA solution for 30 min in the dark at 28.5°C. Afterward, the embryos were rinsed with E3 media and imaged with (SparkCyto, TECAN). The fluorescence intensity of individual larva was quantified using the Image J program (v1.8.0).

### MDA Analysis

Four days post fertilization embryos were collected and grinded manually in PBS. The level of MDA was analyzed by the MDA Detection Kit (S0131, Beyotime, China), and measured by Tecan Infinite M1000 PRO microplate reader (Tecan, Switzerland). At least 30 embryos were measured as one biological repeat, and three biological repeats were performed for each treatment condition.

### mRNA Extraction and QPCR

Total RNA of zebrafish embryos was extracted by a RNA-Quick Purification Kit (RN001, ES Science), and then converted to single strand cDNA with HiFiScript cDNA Synthesis Kit (CW2569M, CWBIO). Real-time PCR was performed using the two-step quantitative RT-PCR method with 2 X SYBR Green qPCR Mater Mix (B21202, Bimake). The sequences of all primers used in the study are listed in Supplementary Table S1.

### Statistics

All data are presented as the mean ± the standard error of the mean. Differences between two groups were analyzed using the two-tailed Student’s *t*-test. Multiple group comparison was conducted by one-way ANOVA. A *p* value <0.05 was considered as statistically significant. For the test of drug combination effect, the Bliss independent model was used (Bliss, 2008; Foucquier and Guedj, 2015). For each group, the rescue effects E was calculated by (number of RBC/s in the treatment group)/(number of RBC/s in the control group) × 100%. The observed drug combination effect is expressed as *E*_AB_ (0 ≤ *E*_AB_ ≤ 1), and the expected additive effect is represented by the probabilistic independence general formula: *E*_A_ + *E*_B_ (1 − *E*_A_) = *E*_A_ + *E*_B_ − *E*_A_ *E*_B_, where 0 ≤ *E*_A_ ≤ 1 and 0 ≤ *E*_B_ ≤ 1. *t*-test was performed to compare the observed effect with the additive effect, and the *p*-value was shown on corresponding figures. The resulting Combination Index (CI) was also displayed on the figures, which can be calculated as: $\text{CI}=\frac{E_{\text{A}}+E_{\text{B}}-E_{\text{A}}E_{\text{B}}}{E_{\text{AB}}}$. When CI < 1, the combination effect is greater than the expected additive effect.

## Results

### GXNT Restored the Circulation of Erythrocytes and Platelets in PHZ-Stimulated Zebrafish

**FIGURE 1:**
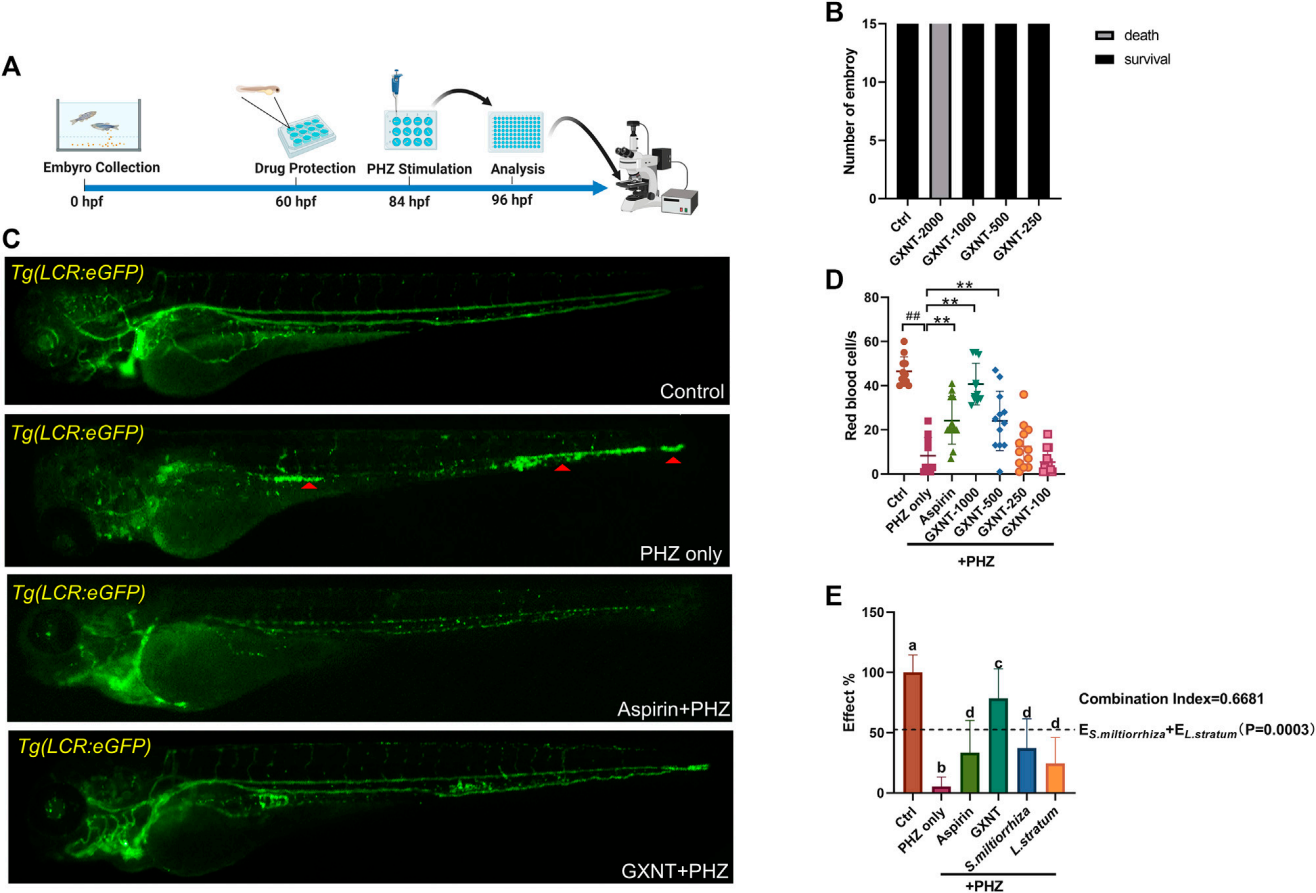
GXNT restored blood circulation in PHZ-stimulated zebrafish thrombosis model. **(A)** Time line of drug protection and PHZ treatment. **(B)** Toxicity effects of different doses of GXNT on fish survival and general development. **(C**,**D)** Representative images **(C)** and quantification **(D)** of erythrocytes circulation in Tg (LCR:eGFP) fish embryos with different treatment. The concentration for the GXNT group was 1 mg/ml. Red triangles marked erythrocytes aggregation. ^#^Compared with the control group; *compared with the model group; ^##^ or **p < 0.01. **(E)** Comparison of the therapeutic effects in blood circulation between embryos treated with single herb and GXNT. Groups labeled without a common letter were significantly different (*p* < 0.05). The *p*-value of *t*-test comparing the observed effect with the additive effect is shown on **(E)**.

**FIGURE 2:**
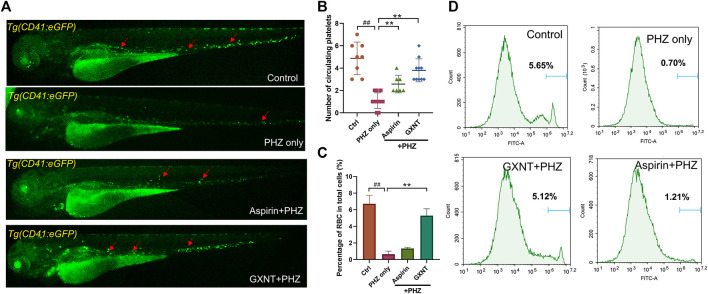
GXNT restored platelet circulation and inhibited hemolysis in PHZ-stimulated zebrafish thrombosis model. **(A**,**B)** Representative images **(A)** and quantification **(B)** of platelets circulation in Tg (CD41:eGFP) fish embryos with different treatment. Red arrows marked circulating platelets. **(C**,**D)** Percentage of GFP labeled erythrocytes of Tg (LCR:eGFP) fish embryos with different treatment. ^#^Compared with the control group; *compared with the model group; ^##^ or **p < 0.01.

As previously reported, the peripheral blood flow was gradually slowed down and become obviously obstructed in the embryos of erythrocyte-labelled *Tg* (*LCR:eGFP*) transgenic zebrafish lines after 12 h incubation in 0.75 μM PHZ solution (Figure 1C; Supplementary Videos S1, S2), indicating thrombi formation, so we continued to examine the efficacy of GXNT on this model. Toxicity analysis suggested that within a range of 100 μg/ml to 1 mg/ml, GXNT incubation from 60 to 84 hpf showed no observable effect on the survival or general development of zebrafish embryos, so the maximum concentration for subsequent studies was set to be 1 mg/ml (Figure 1B). Next, we added the 24-h GXNT treatment step before PHZ stimulation, and evaluated its effects on endogenous blood circulation (Figure 1A). Through quantification analysis of erythrocyte movement based on fluorescent imaging, a significant restoration of blood flow was observed in the embryos protected by GXNT in a dose-dependent manner, with 1 mg/ml concentrationproviding the best rescue effects, and 30 μg/ml aspirin was used as a positive drug (Figures 1C,D; upplementary Videos S3, S4). In order to inquire whether platelets also participate in PHZ-induced haemostasis, the effect of GXNT was tested in the platelet-labelled *Tg* (*CD41:eGFP*) transgenic line. Noticeably, the number of circulating platelets was largely reduced after PHZ stimulation, but was strongly recovered after GXNT or aspirin treatment (Figures 2A,B; Supplementary Videos S6–S9), suggesting that platelet activation and aggregation were also involved in PHZ-induced thrombogenesis, and GXNT may negatively regulate these processes.

Aside from being a haemostasis inducer, PHZ has a well-established role in haemolysis caused by the oxidative damage to erythrocytes membrane (Chen and Collier, 1951). Indeed, considerable reduction in the number of erythrocytes was detected in PHZ treated embryos, and aspirin treatment failed to rescue the process. Interestingly, GXNT protection dramatically inhibited the haemolytic phenotype caused by PHZ, suggesting the multi-target effects of GXNT (Figures 2C,D).

### *S. miltiorrhiza* and *L. striatum* Treatment Alone Showed Reduced Anti-thrombotic Effects

GXNT is consisted of extracts from two naturally derived herbs, *S. miltiorrhiza* and *L. stratum*, according to the traditional drug compatibility theory of TCM. Despite positive clinical results were reported for GXNT in the treatment of thrombotic diseases (Sun et al., 2019), whether its effect does require the combination of both herbs remains unclear from the view of modern science. In order to test the validity of this drug combination, we compared the separate effects of *S. miltiorrhiza* and *L. stratum* single treatment side-by-side with GXNT in our model system. The respective dosage of the single herb extract used in the comparison test was consistent with its respective contents in the GXNT total extract. Markedly, compared to the robust capability of GXNT in restoring the blood flow in PHZ-stimulated embryos, *S. miltiorrhiza* and *L. stratum* only have a mild to medium level anti-thrombotic effect when used by itself (Figure 1E). Moreover, statistical significance was detected between GXNT’s effect and the additive effects of *S. miltiorrhiza* and *L. stratum*, which indicated the existence of synergistic effects between the two herbs. Thus, these results provided biological supports regarding to the combinational usage of *S. miltiorrhiza* and *L. stratum* in the regulation of thrombosis.

### Characterization of Chemical Constituents in GXNT and Its Composing Herbs

**FIGURE 3:**
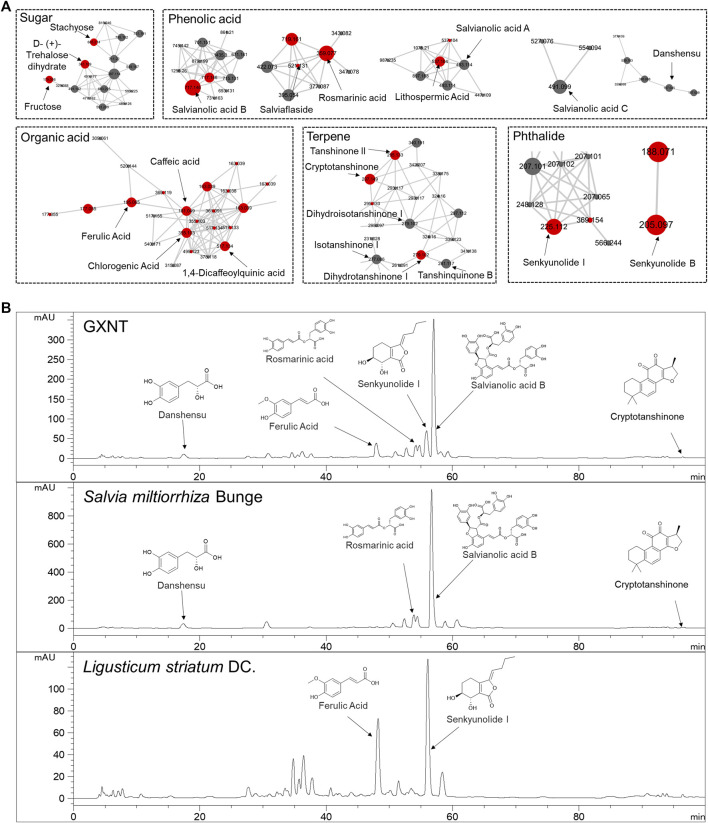
Characterization of the chemical composition in GXNT. **(A)** Molecular networking of GXNT, the main clusters consist of sugar, phenolic acid, terpene. GNPS library annotated nodes are represented in red. **(B)** HPLC chromatogram of GXNT, *Salvia miltiorrhiza* Bunge and *Ligusticum striatum* DC.

Next, in order to inquiry what the chemical compounds are that mediate the anti-thrombotic effects of GXNT and its composing herbs, the main chemical constituents in GXNT were analysed by mass spectrometry. A rapid profiling of GXNT was performed by high resolution mass spectrometry (HRMS) on both positive and negative modes, and the mass data was processed by MZmine2 software to recognize individual ion peaks and the corresponding MS/MS data. Subsequently, the molecular networking of GXNT was created using the processed data (Figure 3A). After GNPS spectral libraries searching and literatures comparison based on the m/z and predicted molecular formula, a total of 43 compounds were temporary identified (Supplementary Figure S1, Table S2). The compounds of sugar, phenolic acid and terpene constructed the main clusters.

To further determine the composition and herbal origins of these compounds in GXNT, we optimized the chromatographic conditions on HPLC-MS for better separation, and analysed GXNT, together with its composing herbs *S. miltiorrhiza* and *L. stratum* (Figure 3B). As a result, danshensu, protocatechualdehyde, rosmarinic acid, lithospermic acid, salvianolic acid B, salvianolic acid A, salvianolic acid C are main GXNT compounds originated from *S. miltiorrhiza*, while senkyunolide B, chlorogenic acid, cryptochlorogenic acid, caffeic acid, ferulic acid, senkyunolide I, ligustilide are identified from *L. stratum*. Based on the results, we selected five compounds (danshensu, ferulic acid, rosmarinic acid, senkyunolide I, salvianolic acid B) of the major peaks and crytotanshinone (based on our previous findings (Sheng et al., 2020) for standard reference comparison and further study (Supplementary Figure S2).

### *In Vivo* Screening Identified the Synergistic Anti-Thrombotic Effects Between Senkyunolide I and Cryptotanshinone

**FIGURE 4:**
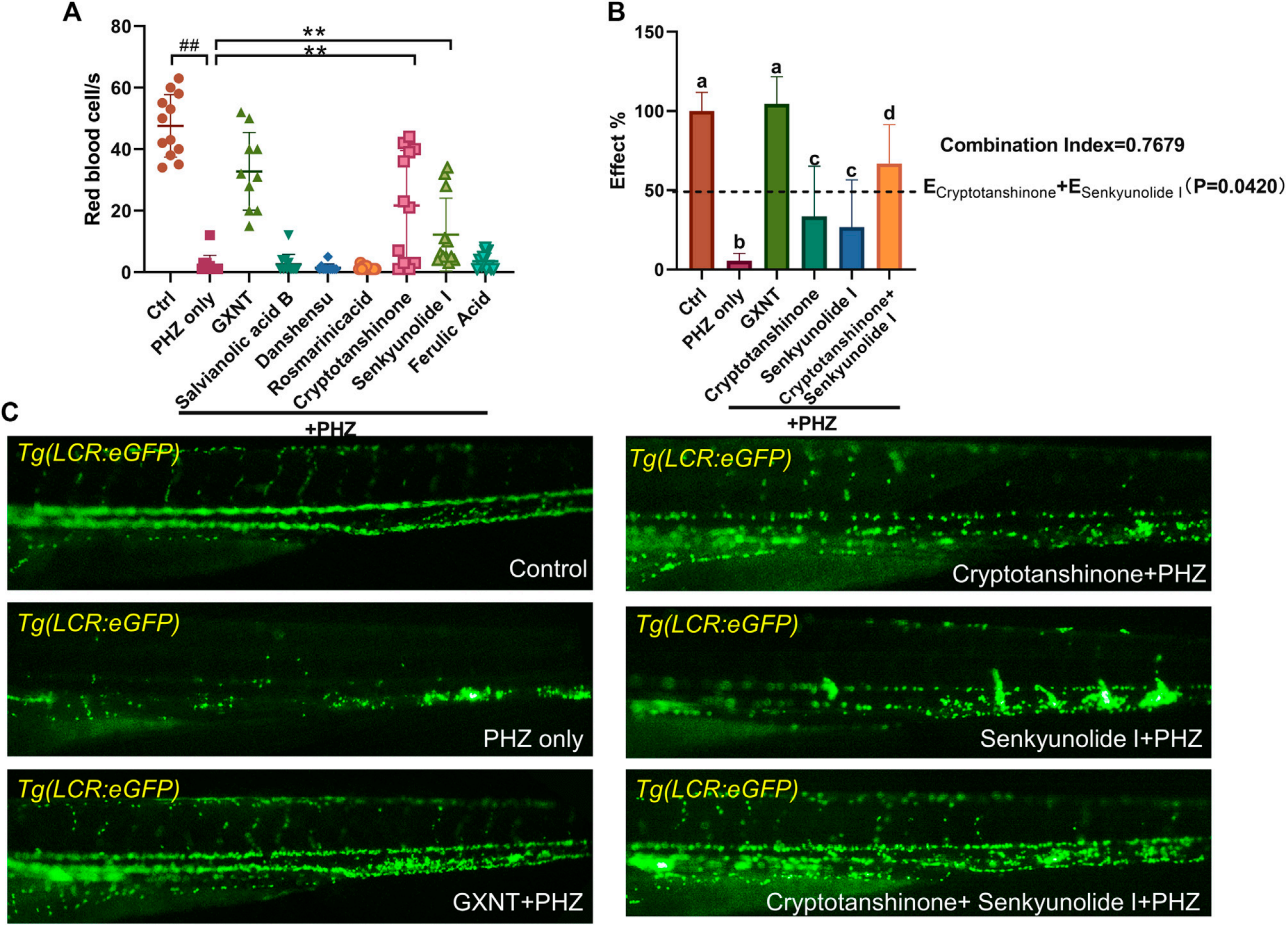
Cyptotanshinone and senkyunolide I showed synergistic anti-thrombotic effects in PHZ-stimulated zebrafish. **(A)** Quantification of erythrocytes circulation in Tg (LCR:eGFP) fish embryos treated with different compounds. ^#^Compared with the control group; *compared with the model group; ^##^ or **p < 0.01. **(B**,**C)** Representative images **(C)** and quantification **(B)** of erythrocytes circulation in Tg (LCR:eGFP) fish embryos with labeled treatment. Groups labeled without a common letter were significantly different (*p* < 0.05). The *p*-value of *t*-test comparing the observed effect with the additive effect is shown on **(B)**.

We continued to screen for the active GXNT compounds with anti-thrombotic effects in the zebrafish thrombosis model. The endogenous blood flow was used as the readout. Notably, senkyunolide I and cryptotanshinone were capable to significantly suppress PHZ-induced thrombogenesis, whereas the rest five compounds failed to display any detectable effects when used alone (Figure 4A). Since cryptotanshinone is a representative compound in *S. miltiorrhiza*, and senkyunolide I is primarily derived from *L. stratum*, we inquired if any cooperative effect exists between the two compounds, which may contribute to the synergistic effects of the two plants that we observed earlierly. Thus, the effects of combined usage of senkyunolide I and cryptotanshinone were examined and compared side-by-side with the effects of single administration. As anticipated, when used together, senkyunolide I and cryptotanshinone showed further increased effects in anti-thrombosis than that of each single compound, to a larger degree than simply additive effects (Figures 4B,C; Supplementary Video S5). Therefore, these results suggested that senkyunolide I and cryptotanshinone could be the key chemical compounds which drives the synergistic anti-thrombotic effects between *S. miltiorrhiza* and *L. stratum* in GXNT. Nevertheless, it is worth noticing that the anti-thrombotic effect of GXNT is still superior than the combined usage of cryptotanshinone and senkyunolide I, suggesting that there remain other unidentified compounds contributing to the total effect.

### GXNT and Cryptotanshinone Inhibit PHZ-Induced Oxidative Damage

**FIGURE 5:**
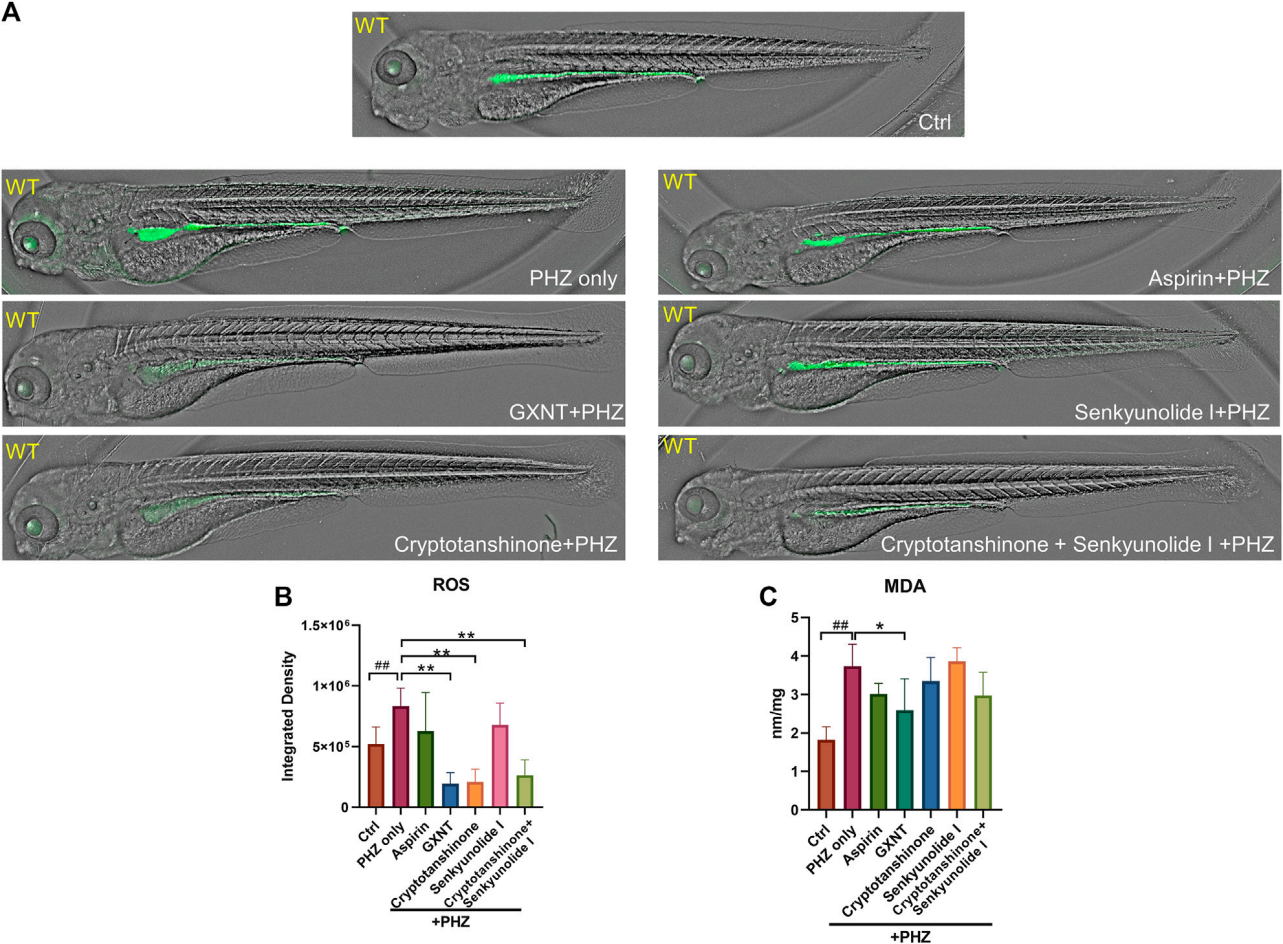
GXNT and cryptotanshinone inhibits oxidative damages in PHZ-stimulated zebrafish. **(A**,**B)** Representative images **(A)** and quantification **(B)** of the fluorescence signals of DCFH-DA probe in 3.5 dpf control embryos or PHZ treated embryos with or without drug protection. **(C)** Concentration of MDA in embryos with different treatment. ^#^Compared with the control group; *compared with the model group; *p < 0.05, ^##^ or **p < 0.01.

The oxidation of PHZ leads to the production of superoxide radicals, hydrogen peroxide, and other oxidative substances (Misra and Fridovich, 1976), which accumulation may lead to oxidative damage to the membrane of endothelial and red blood cells, eventually results in thrombosis and hemolysis. Thus, the impact of GXNT and its components on the endogenous oxidative stress after PHZ stimulation was examined by measuring the levels of free radicals and lipid peroxidation. As observed in previous studies, a moderate level of fluorescent signal was detected in the intestinal region of untreated zebrafish embryos with the ROS probe DCFH-DA, which possibly reflects the physiological level of oxidative stress during normal intestinal development (Shi et al., 2014). The endogenous ROS signal was significantly increased after PHZ incubation, whereas GXNT protection greatly suppressed it to an even lower level than control embryos, supporting its strong anti-oxidative effect (Figures 5A,B). Interestingly, cryptotanshinone protection alone was sufficient to inhibit the ROS level to a comparable degree as GXNT, yet senkyunolide I treatment failed to show any effect, suggesting that the regulation of GXNT on oxidative stress may be primarily derived from the cryptotanshinone, instead of senkyunolide I. We further examined the impact of these drugs on lipid peroxidation by maldondialdehyde (MDA) analysis. The endogenous concentration of MDA increased after PHZ stimulation, and GXNT pre-incubation can effectively suppress its elevation (Figure 5C). Cryptotanshinone treatment also showed a tendency of MDA inhibition. Taken together, these results suggested that GXNT and its component cryptotanshinone possess strong anti-oxidative effects, which may be related to their roles in thrombosis regulation.

### Cryptotanshinone and Senkyunolide I Showed Regulatory Effects on Platelet Activation and Coagulation Cascade

**FIGURE 6:**
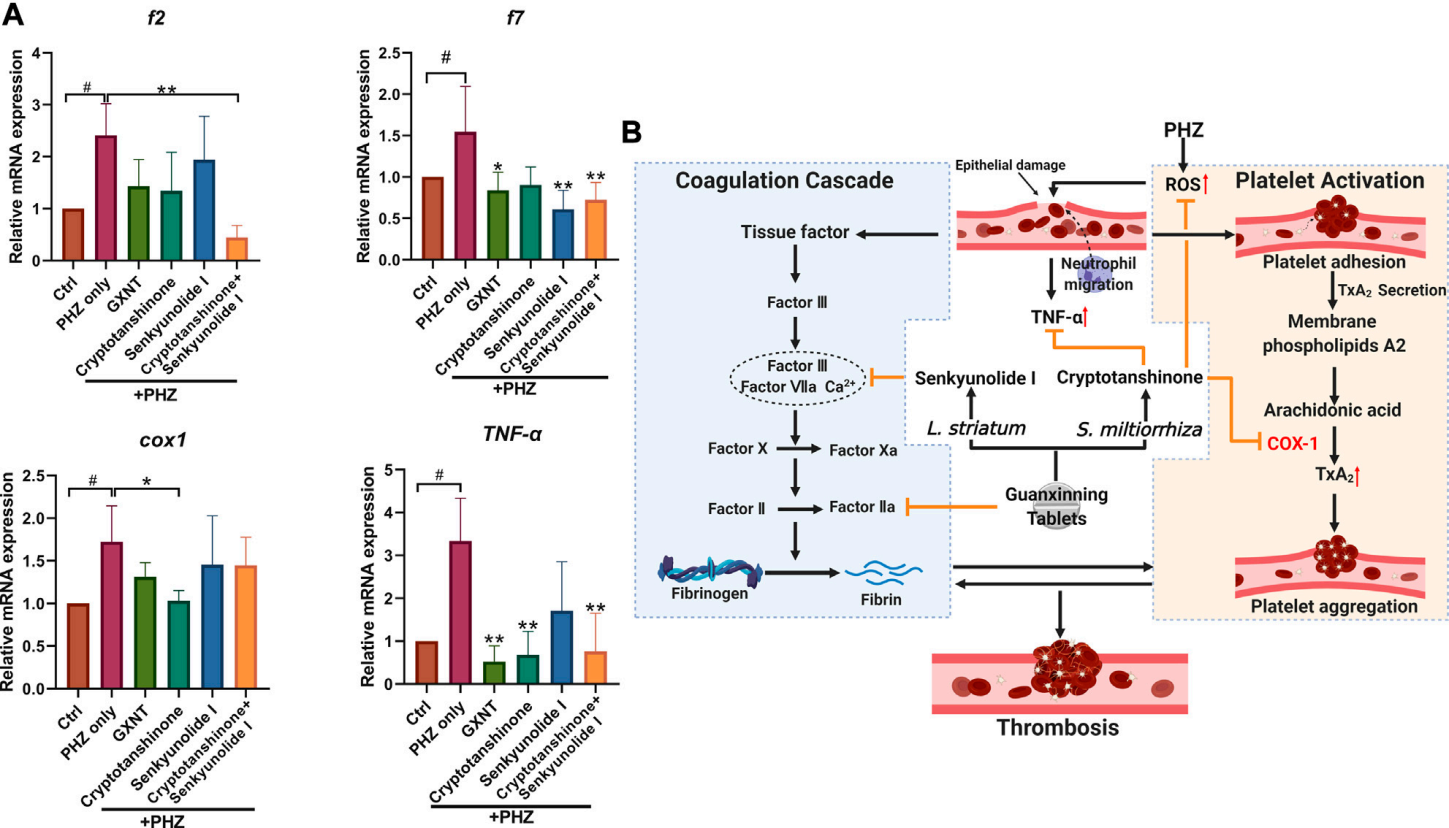
Expression of representative factors in coagulation cascade and platelet activation in PHZ-stimulated zebrafish. **(A)** Expression of coagulation and platelet activation related factors with significant alterations in embryos with GXNT or compound treatment, compared with PHZ only stimulated group. **(B)** A proposed model showing the synergistic mechanism of GXNT and its major components in thrombosis regulation (created with BioRender.com).. The complete set of data are shown in Supplementary Figure S3. ^#^Compared with the control group; *compared with the model group; ^#^ or *p < 0.05, ^##^ or **p < 0.01.

Finally, we investigated the influences of GXNT and its effective components on the processes of platelet activation and coagulation cascade (Figure 6; Supplementary Figure S3). The endogenous transcriptional levels of key factors in the two pathways were examined, and multiple genes showed altered expression after PHZ stimulation. Consistent with our previous findings in *Tg* (*CD41:eGFP*) fish, increased expression of *cox1* was detected in PHZ treated embryos, suggesting the role of platelet activation in our thrombosis model. Noticeably, cryptotanshinone treatment was sufficient to inhibit *cox1* expression. Besides, the expression of tumor necrosis factor α (*tnf*-α), a proinflammatory cytokine related to platelet hyperactivity, was also increased in our disease model, and was substantially inhibited by cryptotanshinone and GXNT. In the coagulation cascade, senkyunolide I was able to significantly inhibit the expression of coagulation factor VII (*f7*), which is a key component of the extrinsic factor X activating complex and is critical for the coagulation cascade in vascular endothelial injury. Downregulated expression of fibrinogen beta chain (*fgb*) was also observed in senkyunolide I protected embryos. Moreover, dramatically decreased expression of coagulation factor II (*f2*, or thrombin) was detected in embryos with combined treatment of cryptotanshinone and senkyunolide I. Collectively, these results suggested that cryptotanshinone and senkyunolide I regulated the endogenous platelet activation and coagulation cascade at multiple levels.

## Discussion

Different from Western pharmacological concept, which usually aimed at one compound with one or more identified specific targets, TCM was formulated through a “polypharmacologic” approach (Newman, 2020). A mixture of natural products was selected according to TCM compatibility theory, which may have limited effect when used alone, but showed enhanced or synergistic results with particular combinations. However, due to the extremely complicated chemical composition in naturally derived herbs and other materials, the pharmacological mechanism of most TCM are largely unclear, making it difficult to evaluate the validity of TCM combinations (Yuan and Lin, 2000) Nevertheless, as many single-targeted chemical drugs frequently encountered bottlenecks in the treatment of complex diseases, such as drug resistance or intolerance, the importance of multi-target and multi-pathway therapeutic strategies has been proposed in recent years (Keith et al., 2005). Re-examining classical TCM formulae, which are excel in using drug combinations, may bring us new hint.

*S. miltiorrhiza* and *L. striatum* are probably among the most widely used herbs with documented function in promoting blood flow, and were used in the treatment of blood stasis symptoms (primarily cardiovascular diseases) in China and some other Asian countries (Wang et al., 2017) A study in 5,284 hospitalized CAD patients in Beijing and Tianjin found that *S. miltiorrhiza* was prescribed to 63.10% of all patients, while *L. striatum* was administered to 46.36% of the patients (Gao et al., 2012). According to the TCM theory, the primary function of *S. miltiorrhiz* lies in promoting blood circulation and resolving haemostasis, and *L. striatum* is capable of enhancing energy balance and internal homeostasis. Nevertheless, despite usually prescribed together, how *S. miltiorrhiza* and *L. striatum* cooperate with each other in thrombosis regulation has little biological evidence. At the compound level, a couple of *S. miltiorrhiz* derived substances were suggested in thrombosis regulation by cell or animal studies, such as salvianolic acid A (Huang et al., 2010), salvianolic acid B (Zhang et al., 2020), dihydrotanshinone I (Park et al., 2008) and tanshinone IIA (Wang et al., 2020). Our previous study also detected the anti-thrombotic effects of cryptotanshinone (Sheng et al., 2020). The pharmacological mechanism of *L. striatum* is less examined than *S. miltiorrhiz*. The function of ligustrazine (tetramethylpyrazine) was comparatively well studied, and was indicated in multiple biological processes, including tumor metastasis, neurogenesis and thrombosis (Liu and Sylvester, 1990; Zou et al., 2018). However, ligustrazine was not identified in the *L. striatum* or GXNT extract in our mass spectrometry analysis, may be because of the difference in sample preparation or herbal species difference.

In our study, a new synergistic cooperation between cryptotanshinone and senkyunolide I in inhibiting endogenous thrombosis was proposed. There is still limited knowledge about the anti-thrombotic effects of the two compounds. Cryptotanshione was previously identified as a STAT3 inhibitor with antitumor activity (Shin et al., 2009), and the effects of senkyunolide I was mostly related to neuroinflammation (Han et al., 2018). Our findings suggested that multiple signaling pathways may be involved in the cooperative effects between cryptotanshinone and senkyunolide I in thrombosis. In our disease model, increased level of free radicals generated by PHZ oxidation is likely to be the initial pathological change, which then result in platelets aggregation and hemolysis through membranous damage of vascular endothelium and erythrocytes. Increased oxidative stress was also discovered in the development of venous and arterial thrombi in the human body, possibly through affecting the RBC membrane (Wang and Zennadi, 2020) and platelet activity (Violi et al., 2017), respectively. Cryptotanshinone showed strong inhibition on the endogenous levels of oxidative radicals and lipid peroxidation, which partially explained its anti-thrombotic activity. Besides, increased level of inflammatory cytokine TNF-α was detected in PHZ-stimulated embryos, which was greatly downregulated by cryptotanshinone or GXNT. Previous studies suggested that TNF-α, and other inflammatory cytokines such as IL-17A, may has a role in inducing the formation of neutrophil extracellular traps (NETs) (Khandpur et al., 2013), which is a specific type of cell death involved in many diseases including both VET and atherosclerosis (Mozzini et al., 2017). Whether cryptotanshinone and *S. miltiorrhiza* can regulate NETs release is interesting to test in the future. Moreover, decreased level of COX-1 was also detected in embryos protected with cryptotanshione, suggesting its possible role in platelet activation. On the other hand, the potential mechanism of senkyunolide I in thrombosis tends to be more related to the coagulation cascade. Both the expression of coagulation factor f7, which is a key factor in the extrinsic coagulation signaling, and the downstream fibrinogen were downregulated by senkyunolide I. Although still rather limited, our results suggested that cryptotanshinone and senkyunolide I may regulate thrombosis at multiple levels in different signaling pathways, which partially explained their synergistic effects.

Finally, there remain some limitations in our study. For example, only six compounds were tested in our model system for their anti-thrombotic effects. By combining the zebrafish model system with high-throughput screening platform, such as high-content analysis, we shall be able to examine the function of compounds of higher order of magnitude in the future. Besides, when testing the expression of downstream factors, whole embryo lysate extracted mRNA was used, which sensitivity can be influenced by cellular heterogeneity. Other experimental techniques, such as cell sorting, single cell sequencing, or transgenic fish reporter lines with specifically labeled downstream target, will be helpful to solve this problem.

Taken together, through zebrafish thrombotic model-based functional screening and high-resolution mass spectrometry, we investigated the effects of herb combinations and compound combinations of GXNT in the treatment of thrombosis. The synergistic effects of *S. miltiorrhiza* and *L. striatum*, as well as of their respective compound, cryptotanshinone and senkyunolide I, were firstly demonstrated in our study. Multiple signaling pathways, including oxidative stress, platelet activation and coagulation cascade were suggested to be regulated differently by these factors. Our study provides solid biological evidence toward the drug combination rationale in a TCM formulae and brings new therapeutic candidates in the management of thrombosis.

## Data Availability Statement

The original contributions presented in the study are included in the article/Supplementary Material, further inquiries can be directed to the corresponding author.

## Ethics Statement

The animal study was reviewed and approved by Animal Ethics Committee of the Laboratory Animal Center, Zhejiang University.

## Author Contributions

Conception and design: LZ and YW. Acquisition of data: JL, HL, and QY. Analysis and interpretation of data: JL, HL, and ZY. Writing of the manuscript: LZ, HL, and JL.

## Funding

This work was supported by National Key Scientific and Technological Project of China (Grant No. 2018ZX09201011), National Natural Science Foundation of China (Grant Nos. 31971088 and 81822047), and Natural Science Foundation of Zhejiang Province (Grant No. LGF21H280005).

## Conflict of Interest

Author QY was employed by the company Chiatai Qingchunbao Pharmaceutical Co., Ltd.

The remaining authors declare that the research was conducted in the absence of any commercial or financial relationships that could be construed as a potential conflict of interest.

## Abbreviations

CAD, coronary artery disease; CI, combination index; COX1, cyclooxygenase-1; fgb, fibrinogen beta chain; GXNT, guanxinning tablet; HRMS, high resolution mass spectrometry; MDA, maldondialdehyde; NETs, neutrophil extracellular traps; PHZ, phenylhydrazine; PTGS1, prostaglandin-endoperoxide synthase one; TCM, traditional Chinese medicine; tnf-α, tumor necrosis factor α; TxA2, thromboxane A2; VET, venous thromboembolism.
