# Supplementary material for: Synergistic Effects of Cryptotanshinone and Senkyunolide I in Guanxinning Tablet Against Endogenous Thrombus Formation in Zebrafish
Source: Front Pharmacol. 2021 Jan 14;11:622787. doi: 10.3389/fphar.2020.622787 (PMC7841298; doi:10.3389/fphar.2020.622787)
Supplement: Supplementary file 10 [file datasheet1.docx]

Supplementary Material

# Supplementary Figures


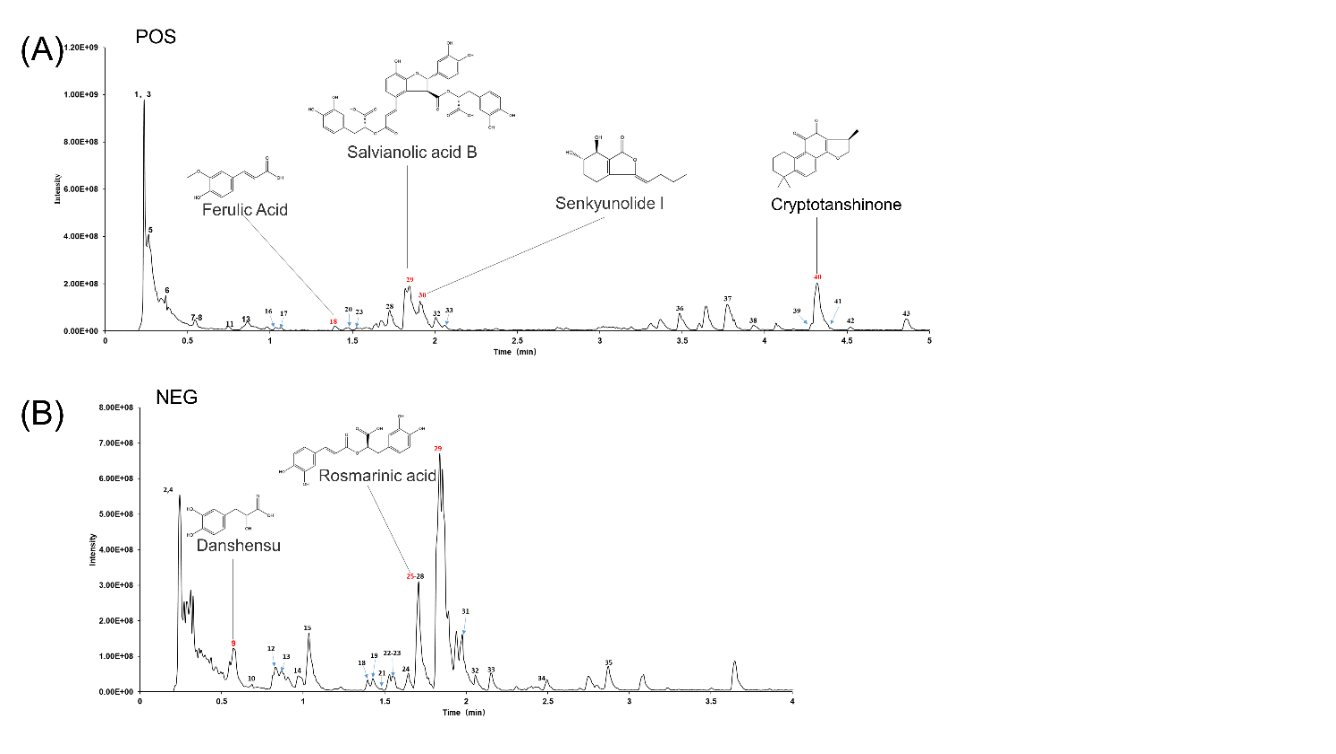


**Supplementary Figure 1.** **Base peak chromatogram of GXNT obtained by UPLC-Q-TOF in positive (A) and negative (B) ion modes. Peaks are numbered according to Supplementary Table 2.**


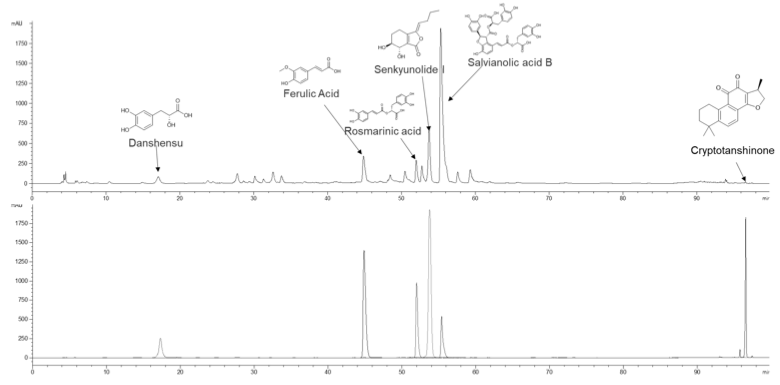


**Supplementary Figure 2.** HPLC chromatogram of GXNT (up panel) and standard comparison of danshensu, ferulic acid, rosmarinic acid, senkyunolide I, salvianolic acid B and cryptotanshinone (down panel).

**
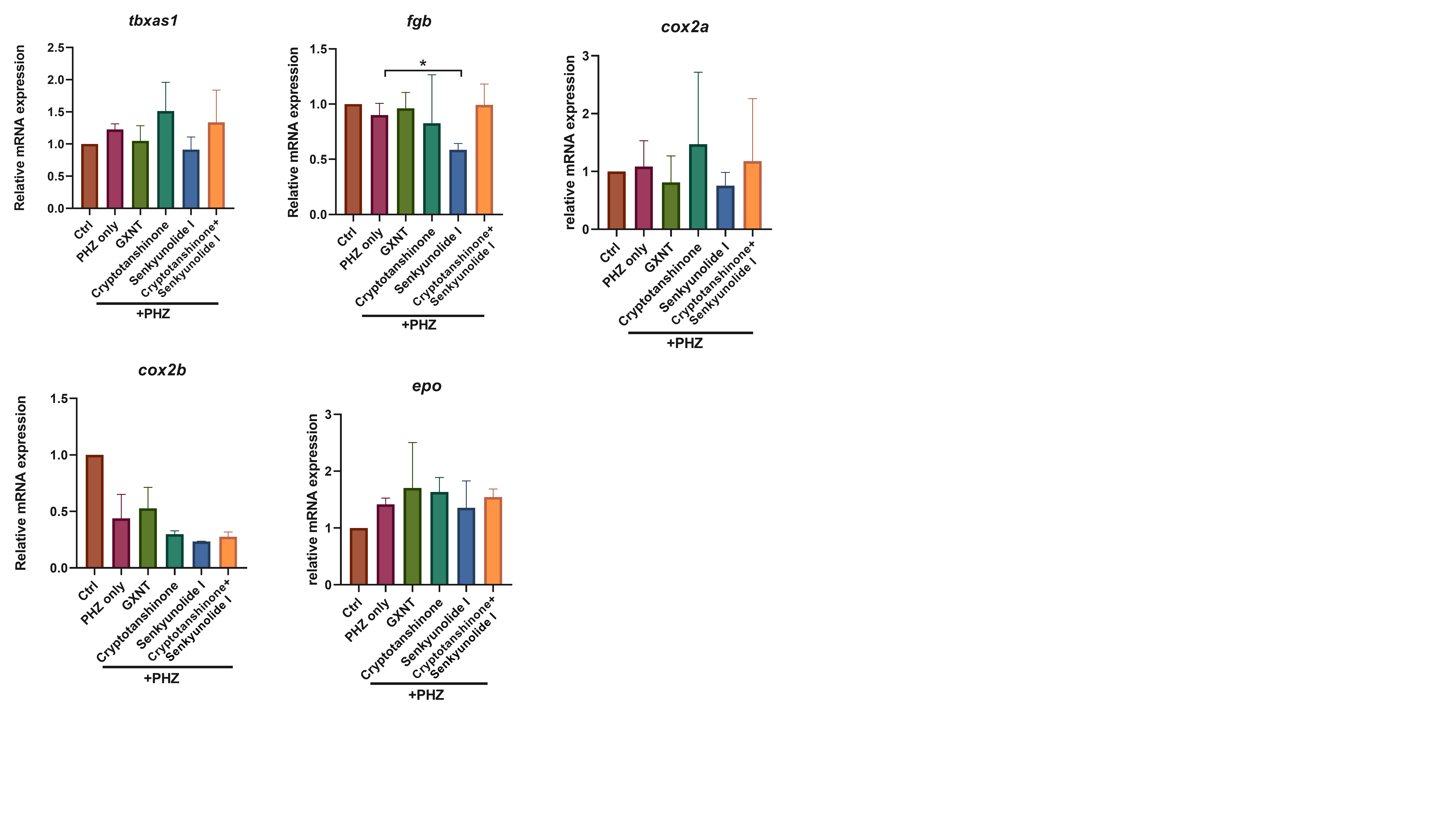
**

**Supplementary Figure 3.** **Expression of representative factors in coagulation cascade and platelet activation in embryos with different treatment. *compared with the model group; *, *p* < 0.05.**

# Supplementary Tables

**Supplementary Table 1.** List of **QPCR primer sequences used in this study.**

| Gene name | Forward primer | Reverse primer |
| --- | --- | --- |
| *f2* | ACAACTGGAAGGAAAACCTGAA | ACGGCCCTTATAACCTGCAAA |
| *f7* | GGTGAGAAGGGTTTCTGTGGA | CCACCTCCAGATCATGCTCAC |
| *fga* | CATTCACTGCTCTGCCTGTTC | CGCCTCTAGGGTTCACCAC |
| *cox1* | CTCGGCATGTACGCTACCTT | TAATGATGAGCCTGGCGGTC |
| *cox2a* | TCCACAGAGGAGCAGTCTCA | AATGTGCCCCAGATCCACTC |
| *cox2b* | TTGTTGCTCCCATCCCTGTC | GAAACTCGGGTGTTGTGCAG |
| *tbxas1* | GAGCACTGGACTGAACCCAC | CGCTCCAAACGGCAGATACA |
| *epo* | AGGAGGCAGGATATGGACTATTAC | ACAGTTGGAGGTGCTTGAGG |
| *TNF-α* | ACCAGGCCTTTTCTTCAGGT | TTTGCCTCCGTAGGATTCAG |
| *ef1a* (internal reference） | AGAAGGCTGCCAAGACCAAG | AGAGGTTGGGAAGAACACGC |

**Supplementary Table 2.** Characterization of constituents in GXNT by UPLC-Q-TOF.

| No. | RT  (min） | Detected m/z | Molecular Formula | Identification | References |
| --- | --- | --- | --- | --- | --- |
| 1 | 0.22 | 175.1188[M+H]^+^ | C_6_H_14_N_4_O_2_ | Arginine | (Shao-Jun et al., 2008) |
| 2 | 0.23 | 665.2140[M-H]^-^ | C_24_H_42_O_21_ | Stachyose | (Chen et al., 2011) |
| 3 | 0.23 | 527.1580[M+H]^+^ | C_18_H_32_O_16_ | Maltotriose | (Chen et al., 2011) |
| 4 | 0.24 | 341.1090[M-H]^-^ | C_12_H_26_O_13_ | D- (+)-Trehalose dihydrate |  |
| 5 | 0.29 | 360.1500[M+H]^+^ | C_12_H_22_O_11_ | Palatinose |  |
| 6 | 0.39 | 132.1018[M+H]^+^ | C_6_H_13_NO_2_ | Isoleucine | (Kobayashi et al., 2012) |
| 7 | 0.54 | 328.1392[M+H]^+^ | C_15_H_21_NO_7_ | N-(1-Deoxy-1-fructosyl) phenylalanine | (Kobayashi et al., 2012) |
| 8 | 0.54 | 166.0863[M+H]^+^ | C_9_H_11_NO_2_ | Phenylalanine | (Kobayashi et al., 2012) |
| 9^*^ | 0.57 | 197.0453[M-H]^-^ | C_9_H_10_O_5_ | Danshensu | (Liu et al., 2007) |
| 10 | 0.69 | 353.0879[M-H]^-^ | C_16_H_18_O_9_ | Chlorogenic acid isomer |  |
| 11 | 0.74 | 205.0973[M+H]^+^ | C_12_H_12_O_3_ | Senkyunolide B | (Zhang et al., 2017) |
| 12^*^ | 0.83 | 137.0242[M-H]^-^ | C_7_H_6_O_3_ | Protocatechualdehyde | (Liu et al., 2007) |
| 13 | 0.87 | 353.0879[M-H]^-^ | C_16_H_18_O_9_ | Chlorogenic acid | (Liu et al., 2007) |
| 14 | 0.97 | 179.0347[M-H]^-^ | C9H8O4 | Caffeic acid | (Liu et al., 2007) |
| 15 | 1.03 | 167.0347[M-H]^-^ | C_8_H_8_O_4_ | Quinolacetic acid |  |
| 16 | 1.05 | 231.1263[M+H]^+^ | C_13_H_14_N_2_O_2_ | 1,2,3,4-tetrahydroharmane-3-carboxylic acid |  |
| 17 | 1.07 | 420.1858[M+H] ^+^ | C_18_H_26_O_10_ | Icariside F2 | (Yan et al., 2016) |
| 18^*^ | 1.39 | 195.0653[M+H]^+^ | C_10_H_10_O_4_ | Ferulic acid | (Yan et al., 2005) |
| 19 | 1.43 | 241.1081[M-H]^-^ | C_12_H_18_O_5_ | 3-Butyl-4,5,6,7-tetrahydro-3,6,7-trihydroxy-1(3H)-isobenzofuranone | (Songsong et al., 2015) |
| 20 | 1.46 | 223.0602[M+H]^+^ | C_11_H_10_O_5_ | Isofraxidin | (Yongping et al., 2007) |
| 21 | 1.48 | 521.1311[M-H]^-^ | C_24_H_26_O_13_ | Salviaflaside | (2018) |
| 22 | 1.55 | 417.0828[M-H]^-^ | C_20_H_18_O_10_ | Salvianolic acid D | (Liu et al., 2007) |
| 23 | 1.56 | 515.1199[M-H]^-^ | C_25_H_24_O_12_ | 1,4-Dicaffeoylquinic acid | (Dong et al., 2013) |
| 24 | 1.65 | 717.1464[M-H]^-^ | C_36_H_30_O_16_ | Salvianolic acid B isomer | (Liu et al., 2007) |
| 25^*^ | 1.71 | 359.0770[M-H]^-^ | C_18_H_16_O_8_ | Rosmarinic acid | (Liu et al., 2007) |
| 26 | 1.71 | 719.1610[M-H]^-^ | C_36_H_32_O_16_ | Sagerinic acid |  |
| 27 | 1.73 | 493.1143[M-H]^-^ | C_26_H_22_O_10_ | Isosalvianolic acid A | (Dong et al., 2013) |
| 28 | 1.73 | 539.1174[M+H]^+^ | C_27_H_22_O_12_ | Lithospermic acid | (Hong et al., 2012) |
| 29^*^ | 1.84 | 719.1596[M+H]^+^ | C_36_H_30_O_16_ | Salvianolic acid B | (Liu et al., 2007) |
| 30^*^ | 1.91 | 225.1118[M+H]^+^ | C_12_H_16_O_4_ | Senkyunolide I | (Yan et al., 2005) |
| 31 | 1.98 | 493.1144[M-H]^-^ | C_26_H_22_O_10_ | Salvianolic acid A | (Liu et al., 2007) |
| 32 | 2.05 | 551.1201[M-H]^-^ | C_28_H_24_O_12_ | Schizotenuin F **or**  3-(3,4-Dihydroxyphenyl)-2-[I-3-[2-(3,4-dihydroxyphenyl)-7-hydroxy-3-methoxycarbonyl-2,3-dihydro-1-benzofuran-4-yl] prop-2-enoyl] oxypropanoic acid | (Hong et al., 2012) |
| 33 | 2.15 | 491.0989[M-H]^-^ | C_26_H_20_O_10_ | Salvianolic acid C | (Liu et al., 2007) |
| 34 | 2.49 | 313.0719[M-H]^-^ | C_17_H_14_O_6_ | Nepetoidin A |  |
| 35 | 2.87 | 205.0869[M-H]^-^ | C_12_H_14_O_3_ | 3-Butyl-4-hydroxy-1(3H)-isobenzofuranone | (Zhang et al., 2017) |
| 36 | 3.48 | 288.2898[M+H]^+^ | C_17_H_37_NO_2_ | 2-Amino-1,3-heptadecanediol |  |
| 37 | 3.77 | 279.1021[M+H]^+^ | C_18_H_14_O_3_ | Dihydroisotanshinone I | (Liu et al., 2007) |
| 38 | 3.93 | 281.1174[M+H]^+^ | C_18_H_16_O_3_ | Methylenedihydrotanshinquinone **or** Tanshinquinone B | (Xue et al., 2014) |
| 39 | 4.29 | 277.0861[M+H]^+^ | C_18_H_12_O_3_ | Isotanshinone I | (Ge et al., 2019) |
| 40^*^ | 4.32 | 297.1488[M+H]^+^ | C_19_H_20_O_3_ | Cryptotanshinone | (Liu et al., 2007) |
| 41 | 4.38 | 301.2163[M+H]^+^ | C_20_H_30_O_2_ | (1R,7R)-7-ethenyl-1,4a,7-trimethyl-3,4,4b,5,6,9,10,10a-octahydro-2H-phenanthrene-1-carboxylic acid |  |
| 42 | 4.52 | 279.1017[M+H]^+^ | C_18_H_14_O_3_ | Dihydrotanshinone I | (Liu et al., 2007) |
| 43 | 4.86 | 295.1332[M+H]^+^ | C_19_H_18_O_3_ | Tanshinone II | (Liu et al., 2007) |

**References in Supplementary Table 2**

(2018). Rapid profiling of polymeric phenolic acids in Salvia miltiorrhiza by hybrid data‐dependent/targeted multistage mass spectrometry acquisition based on expected compounds prediction and fragment ion searching. *Journal of Separation Science* 41(8).

Chen, X., Lou, Z., Zhang, H., Tan, G., Liu, Z., Li, W., et al. (2011). Identification of multiple components in Guanxinning injection using hydrophilic interaction liquid chromatography/time‐of‐flight mass spectrometry and reversed‐phase liquid chromatography/time‐of‐flight mass spectrometry. *Rapid Communications in Mass Spectrometry* 25(11).

Dong, J., Zhu, Y., Gao, X., Chang, Y., Wang, M., and Zhang, P. (2013). Qualitative and quantitative analysis of the major constituents in Chinese medicinal preparation Dan-Lou tablet by ultra high performance liquid chromatography/diode-array detector/quadrupole time-of-flight tandem mass spectrometry. *J Pharm Biomed Anal* 80(Complete)**,** 50-62.

Ge, P., Wen, L., Wang, X., Zhang, J., and Xu, G. (2019). Rapidly identify compounds from danshen by using ultra-high-performance liquid chromatography coupled with linear ion trap-Orbitrap mass spectrometer and predict its mechanisms of intervening thrombotic diseases. *Journal of Liquid Chromatography & Related Technologies***,** 1-7.

Hong, Chen, Qin, Zhang, Xiaoming, Wang, et al. (2012). Qualitative analysis and simultaneous quantification of phenolic compounds in the aerial parts of Salvia miltiorrhiza by HPLC-DAD and ESI/MS(n). *Phytochemical analysis : PCA*.

Kobayashi, S., Nagasawa, S., Yamamoto, Y., Donghyo, K., Bamba, T., and Fukusaki, E. (2012). Metabolic profiling and identification of the genetic varieties and agricultural origin of Cnidium officinale and Ligusticum chuanxiong. *Journal of Bioence and Bioengineering* 114(1).

Liu, A.H., Lin, Y.H., Yang, M., Guo, H., Guan, S.H., Sun, J.H., et al. (2007). Development of the fingerprints for the quality of the roots of Salvia miltiorrhiza and its related preparations by HPLC-DAD and LC-MS(n). *J Chromatogr B Analyt Technol Biomed Life Sci* 846(1-2)**,** 32-41. doi: 10.1016/j.jchromb.2006.08.002.

Shao-Jun, L.I., Zhi-Ming, S., Liang-Zhu, W., Zong-Suo, L., and Liang-Liang, F.U. (2008). Protein and Fattiness Evaluation of Salvia miltiorrhiza Seed. *Acta Botanica Boreali-Occidentalia Sinica* 28(9)**,** 1899-1903.

Songsong, W., Haiyu, X., Yan, M., Xuguang, W., Yang, S., Bin, H., et al. (2015). Characterization and rapid identification of chemical constituents of NaoXinTong capsules by UHPLC-linear ion trap/Orbitrap mass spectrometry. *Journal of Pharmaceutical & Biomedical Analysis* 111**,** 104-118.

Xue, Y., Wu, Y., Zhu, H., Li, X.N., Qian, J.F., Lai, Y., et al. (2014). Salviprzols A and B, C21- and C22-terpenoids from the roots of Salvia przewalskii Maxim. *Fitoterapia* 99**,** 204-210.

Yan, Shi-Li, Yan-Fang, and Le-Jie (2016). Three new phthalide glycosides from the rhizomes of Ligusticum chuanxiong. *Phytochemistry Letters*.

Yan, R., Li, S.L., Chung, H.S., Tam, Y.K., and Lin, G. (2005). Simultaneous quantification of 12 bioactive components of Ligusticum chuanxiong Hort. by high-performance liquid chromatography. *Journal of Pharmaceutical & Biomedical Analysis* 37(1)**,** 87-95.

Yongping, L., Xiaomian, N., and Mingfa, W. (2007). Study on Quality Standard for Shenggukang Tablet. *Modern Chinese Medicine*.

Zhang, Q., Wang, M., Wang, Q., Zhao, H., Zhang, Z., Yu, H., et al. (2017). Characterization of the potential new phthalides in Ligusticum chuanxiong Hort. using ultra performance liquid chromatography coupled with quadrupole time of flight tandem mass spectrometry. *Journal of Separation ence* 40(10)**,** NA-NA.

# List of Supplementary videos

# Supplementary Video 1: Blood circulation in an untreated *Tg(LCR:eGFP)* zebrafish embryo.

# Supplementary Video 2: Blood circulation in a PHZ-stimulated *Tg(LCR:eGFP)* zebrafish embryo.

# Supplementary Video 3: Blood circulation in a PHZ-stimulated *Tg(LCR:eGFP)* zebrafish embryo protected with aspirin.

# Supplementary Video 4: Blood circulation in a PHZ-stimulated *Tg(LCR:eGFP)* zebrafish embryo protected with GXNT.

# Supplementary Video 5: Blood circulation in a PHZ-stimulated *Tg(LCR:eGFP)* zebrafish embryo protected with crytotanshinone and senkyunolide I.

# Supplementary Video 6: Platelets circulation in an untreated *Tg(CD41:eGFP)* zebrafish embryo.

# Supplementary Video 7: Platelets circulation in a PHZ-stimualted *Tg(CD41:eGFP)* zebrafish embryo.

# Supplementary Video 8: Platelets circulation in a PHZ-stimulated *Tg(CD41:eGFP)* zebrafish embryo protected with aspirin.

# Supplementary Video 9: Platelets circulation in a PHZ-stimulated *Tg(CD41:eGFP)* zebrafish embryo protected with GXNT.
